# Predictors of quality of family planning counselling in Ethiopia: multilevel analysis of the SPA survey 2021/22

**DOI:** 10.3389/frph.2025.1743257

**Published:** 2026-01-15

**Authors:** Kalayu Brhane Mruts, Tesfay Brhane Gebremariam, Amanuel Tesfay Gebremedhin

**Affiliations:** 1 School of Public Health, Colleges of Health Science, Debre Berhan University, Debre Berhan, Ethiopia; 2 Health Measurement and Evaluation, Strategy and Performance, Murray Primary Health Network, Bendigo, VIC, Australia; 3 School of Nursing and Midwifery, Edith Cowan University, Joondalup, WA, Australia; 4 Curtin School of Population Health, Faculty of Health Sciences, Curtin University, Bentley, WA, Australia; 5 Nutrition and Health Innovation Research Institute, School of Medical and Health Sciences, Edith Cowan University, Perth, WA, Australia

**Keywords:** family planning, contraception, counselling, quality, Ethiopia, SPA

## Abstract

**Background:**

High-quality family planning (FP) counselling is essential for informed contraceptive choice, reducing discontinuation, and improving reproductive health. However, many women in sub-Saharan Africa continue to face barriers. This study examined client-, provider-, and facility-level determinants of FP counselling quality in Ethiopia using nationally representative, multi-source data.

**Methods:**

We analysed data from the 2021/22 Ethiopia Service Provision Assessment, including 2,224 women who received or were prescribed injectables, pills, or implants. Counselling quality was assessed using a 15-item checklist derived from direct observations and client exit interviews. Multilevel ordinal logistic regression was applied to identify predictors at the client-, provider-, and facility-levels.

**Results:**

Only 32% of clients received high-quality FP counselling, with key information on side effects, STI protection, and a follow-up frequently omitted. High-quality counselling was more likely among women who had never used FP (aOR 1.40; 95% CI: 1.01–1.95) or were past users (aOR 2.05; 95% CI: 1.44–2.92), those counselled by providers with more than five years of experience (aOR 1.91; 95% CI: 1.26–2.89), and those served by providers with high adherence to medical-eligibility screening (aOR 1.67; 95% CI: 1.22–2.29). The presence of a FP-trained provider and national FP guidelines was also positively associated. Marked regional disparities were observed, with facilities in Sidama performing better and those in emerging regions lagging behind. Facility-level factors accounted for approximately 52% of the variation in counselling quality.

**Conclusion:**

The quality of FP counselling in Ethiopia remains suboptimal and is largely driven by provider and facility-level factors. Strengthening provider training, ensuring guideline availability, addressing regional disparities, and improving counselling for current users are essential for enhancing service quality, reducing discontinuation, and improving reproductive health outcomes.

## 1 Introduction

Family planning (FP) is a proven strategy for improving maternal and child health and survival, as well as empowering women to participate more fully in education and economic activities (1–3). High-quality FP counselling plays a critical role in facilitating informed contraceptive choices, increasing client satisfaction and promoting method uptake and continued use, and thereby contributing to better reproductive health outcomes (4, 5). Effective counselling enables women and couples to make decisions that align with their reproductive intentions by using appropriate methods, reducing unintended pregnancies and increasing birth spacing. This, in turn, lowers maternal and child mortality while fostering overall family wellbeing (4, 6, 7).

Quality counselling also supports the appropriate selection and consistent use of contraceptive methods, which can minimise discontinuation and method-related complications (4, 8). Providing comprehensive information, especially regarding potential side effects, is essential for helping clients in making informed decisions and continuing method use (8–10). In addition to information exchange, high-quality counselling should ensure that clients are treated with dignity and respect, and that their privacy and confidentiality are maintained throughout the interaction (11).

In recognition of the importance of effective counselling, the World Health Organization (WHO) identifies high-quality FP counselling as a cornerstone for increasing contraceptive use and reducing discontinuation. The WHO emphasises that quality counselling should involve accurate information provision, respect for client preferences, and a supportive, client-centred environment. In alignment with these global standards, the Ethiopian Federal Ministry of Health has revised its national FP guideline to promote an interactive decision-making process between the client and provider. The guideline encourages healthcare providers to deliver comprehensive, unbiased counselling that enables clients to make informed and voluntary decisions about their contraceptive options (12).

Despite such recommendations, women in low- and middle-income countries, particularly in sub-Saharan Africa (SSA), often encounter barriers in accessing high-quality FP counselling (13–15). Existing studies have highlighted numerous factors associated with poor counselling, with most focused on individual-level (13, 14, 16, 17). For instance, studies in Kenya and Ethiopia found that variables such as age, education, marital status and FP history were associated with good quality of FP counselling (13, 14, 16, 17). Although some studies have examined provider-related factors (e.g., experience, workload) and facility-level aspects (e.g., availability of a private counselling space) (15, 18), these factors have not been well investigated, leaving a gap in understanding the broader structural influence on the quality of FP counselling.

Existing studies on FP counselling quality face challenges related to both measurement and analysis. A widely used measure of quality in the area is the Method Information Index (MII), which typically comprises three or six questions focusing on the information provision aspect of counselling (8, 19, 20). While the MII is simple to administer, it captures only a narrow dimension of counselling quality, overlooking broader elements of effective communication and interaction (8, 19, 20). Additionally, it relies on client self-reports collected sometimes after the consultation (15, 17, 20), which is prone to recall and social desirability biases, particularly in low literacy settings such as Ethiopia (15, 17). In contrast, directly observing provider-client interactions or collecting data through client exit interviews using structured checklists like the Service Provision Assessment (SPA) survey can reduce the recall and social desirability bias (21–24). Additionally, these allow for more comprehensive assessment of FP counselling quality by capturing aspects such as respect, privacy and confidentiality, and method-specific information that are often missed in self-report measures (25). In addition to the measurement challenges, the analysis of FP counselling quality also presents limitations. Many studies fail to account for the multilevel structure of FP service delivery, despite clients often being nested within providers and facilities. As a result, single-level or fixed-effects models that don't consider clustering at the provider or facility level may lead to biased estimates and misinterpretation of findings.

Given these gaps, there is a critical need for studies that integrate comprehensive measurement tools with multilevel analytical approaches to explore the determinants of FP counselling quality. This study aimed to address this need by analysing data from the 2021/22 Ethiopia SPA survey, drawing on multiple data sources, including facility inventories, provider interviews, direct observation of provider-client interactions and client exit interviews. The findings will help inform the development of evidence-based strategies to strengthen FP counselling practices and promote equitable access to quality reproductive health services across Ethiopia.

## 2 Methods

### 2.1 Data source and study setting

This study employed facility-based cross-sectional data from the Ethiopia SPA survey, which was conducted nationwide in 2021-2022 across public and private health facilities, except for the Tigray region, due to the civil war that occurred during the data collection period. At the time of the survey, Ethiopia was administratively divided into ten regional states and two city administrations (Addis Ababa and Dire Dawa) (26).

The SPA survey was designed to provide nationally representative estimates for all health facilities combined, for each of the 11 administrative regions separately, and by facility type at a national level, covering hospitals (both public and private), health centres, clinics, and health posts. A stratified random sample of 1,407 health facilities was selected from a master list of 25,711 functioning facilities. First, categorising facilities within each region achieved stratification according to facility type, and for clinics, further stratification was based on clinic designation (higher, medium, lower, or specialty clinics). Within these strata, facilities were selected using systematic sampling with equal probability. The sample included all hospitals in the country, as they are relatively few in number and play a critical role in the health system. Representative samples of health centres, health posts, and clinics were also selected (26).

The SPA survey utilised multiple data sources, where women who came for FP services were observed during their consultation with the service provider and interviewed at exit. Additionally, service providers were interviewed, including the provider linked with delivering FP services, and all facilities involved in the survey were audited to examine the physical environment, equipment and supplies, staffing, and services offered. Clients were systematically selected for observation, with a maximum of five clients per provider and up to 15 observations per facility. Among 2,931 eligible FP clients, 2,572 (88%) clients were successfully observed and interviewed after completing their consultation with their provider (26).

### 2.2 Sample participants

Of the 2,572 clients observed during consultations with the provider and interviewed at exit, 2,468 (approximately 96%) were either provided or prescribed one of the three most common modern contraceptive methods—injectables, pills or implants. Given that the counselling checklist items, particularly those related to information provision, differ by contraceptive method, this study focused exclusively on these three groups to ensure consistency in assessing counselling quality. Of these, 132 women who visited the facilities intended to discontinue contraceptive use were excluded from this study, as they may not have received comprehensive FP counselling. Among the remaining 2,336 women, 104 participants had missing information, particularly women's education (*n* = 55) and age (*n* = 47), which were excluded from this analysis. Consequently, the final analysis included 2,224 women. The study covered 560 health facilities and 691 providers who were linked to the counselling received by these women.

### 2.3 Data collection

The FP services were one of the three key service areas selected for observation and client exit interviews in the 2021–2022 Ethiopia SPA survey. Data were collected by trained health professionals—primarily nurses, midwives, and health officers—with clinical experience. These professionals received specific training to conduct structured observations and client interviews.

Prior to the main data collection, data collectors completed intensive training, which included classroom sessions, mock interviews, and practical exercises at nearby health facilities. A pre-test was conducted from October 25 to 29, 2021, which helped refine the data collection tools and procedures. The fieldwork was carried out between 1 November 2021 and 30 March 2022, with data collected over three days in hospitals, two days in health centres, and one day in clinics and health posts. Data collection was conducted in four widely spoken languages—Amharic, Afan Oromo, Tigrigna, and Somali—across the surveyed regional states to ensure comprehension across the surveyed areas.

Four tools were used to capture family planning–related information: a health facility inventory, health care provider interviews, observations of FP consultations, and client exit interviews. The health facility inventory assessed the availability of FP methods, teaching aids and models, essential equipment, clinical guidelines, and the presence of FP-trained providers. Health care providers who were present during the data collection period and involved in the direct provision of FP services were interviewed, particularly those observed delivering FP counselling or services to clients (26). The FP counselling sessions were observed using a structured checklist, and exit interviews were conducted with women who received FP consultations on the same day as their visit. These interviews were administered immediately after the consultation to enhance accuracy and minimise recall bias. Further details on the methodology and data collection procedures are available in the full Ethiopia SPA survey report (26).

### 2.4 Variables

#### 2.4.1 Outcome variable

The primary outcome of this study was the quality of FP counselling, assessed using a 15-item checklist adapted from the SPA observation and the exit interview questionnaires. These items were selected based on their relevance in reflecting the quality of FP counselling as supported by previous literature (27, 28). Fourteen items were taken from the observation checklist completed by trained interviewers during the client-provider interaction. An additional item was created from the client exit interview, which asked whether the provider discussed the FP method with the client. This item was coded as a binary (yes/no) variable to match the observation-based items, with at least one method reported as “yes” and no methods reported as “no”. A composite score for the overall quality of FP counselling was computed by summing the responses across binary items, each coded as 0 for “No” and 1 for “Yes”, yielding a total score range of 0–15. This total composite score was then categorised into tertiles representing low, moderate, and high overall quality of FP counselling.

#### 2.4.2 Independent variables

We included client-, provider-, and facility-related independent variables, selected based on theoretical relevance and previous evidence on the quality of FP counselling services (27). Client characteristics included age, marital status, education, parity, and prior FP use. Provider characteristics comprised age, gender, qualification, experience, weekly working hours, supervision status, salary supplements, and adherence to medical eligibility screening protocols. Facility-level factors included type, location, ownership, region, availability of FP guidelines, quality assurance systems, FP-trained staff, and a composite index of facility readiness based on the availability of seven key items supporting FP counselling. A detailed description of the independent variables, including operational definitions and categorisations, is provided in Appendix Table 1.

### 2.5 Statistical analysis

Descriptive statistics, including frequencies and percentages, were used to summarise client, provider, and facility-level variables. Given the hierarchical structure of the data, where clients (level 1) were nested within health facilities (level 2), a multilevel ordinal logistic regression analysis was conducted to examine factors associated with the quality of FP counselling. A two-level regression model was selected because the providers linked to each client closely approximated the number of health facilities, making the facility the more appropriate clustering unit.

Four models were fitted sequentially: an empty (null) model with no predictors to assess the baseline facility-level variance; a model including only client-level variables (model I); a model including only provider- and facility-level variables (Model II); and a full model including client-, provider-, and facility-level factors simultaneously (model III). Multicollinearity among independent variables was assessed using the Variance Inflation Factor (VIF), with values above 10 indicating potential multicollinearity.

Model fit was evaluated by comparing the Akaike Information Criterion (AIC) across models, with lower values indicating better fit. The intra-class correlation coefficient (ICC) was calculated to determine the proportion of total variance in counselling quality attributable to differences between facilities. Fixed effects were reported as adjusted odds ratios (AORs) with 95% confidence intervals (CIs), while random effects were summarised using variance estimates and ICC% values. A p-value of less than 0.05 was considered statistically significant. All statistical analyses were performed using Stata software (29).

## 3 Results

### 3.1 Client, provider, and facility characteristics

A total of 2,224 eligible women who were observed and interviewed at exit and who received pills, injectable or implant contraceptive methods at the time of the survey were included in this study. These clients were linked to 691 providers across 560 health facilities. Most clients were under 30 years of age (74%), the majority were married (92%), 76% had attained at least primary education, and 75% of the clients were current users who visited either to obtain re-supply dose of the same method or to switch to another method.

Among the providers who linked to the provision of these three contraceptive methods, 65% were under 30 years old, over half (53%) held a diploma or were HEWs, and only one-fourth had five years or more of work experience. Three in five (60%) had low adherence to medical eligibility screening. Approximately 82% of clients obtained their contraceptive method from public facilities, and 56% were served at lower-level health facilities. Additionally, approximately two-fifths (37%) of the facilities reported not having the national FP guideline available at the time of the survey (Table 1).

**Table 1:** Client, provider and facility characteristics.

Variables	Categories	*N* (%)
Women age	<25 years	814 (36.6%)
	25–29 years	829 (37.3%)
	30–34 years	386 (17.4%)
	≥35 years	195 (8.8%)
Women's highest level of education attended	No education	535 (24.1%)
	Primary	793 (35.7%)
	Secondary and above	896 (40.3%)
Women's marital status)	Not married	181 (8.1%)
	Married	2,043 (91.9%)
Parity	Nulliparity	287 (12.9%)
	Primiparity	526 (23.7%)
	Multiparity	1,186 (53.3%)
	Grand multiparity	225 (10.1%)
Women family planning status at the beginning of consultation	Current user	1,668 (75.0%)
	Past user	273 (12.3%)
	Never user	283 (12.7%)
Provider age	<30 years	451 (65.3%)
	30–34 years	136 (19.7%)
	≥35 years	104 (15.1%)
Provider gender	Male	265 (38.4%)
	Female	426 (61.6%)
Provider qualification	Doctor	13 (1.9%)
	Degree	310 (44.9%)
	Diploma/HEW	368 (53.3%)
Provider experience	<2 years	279 (40.4%)
	3–5 years	229 (33.1%)
	>5 years	183 (26.5%)
Provider working hours	≤40 hours	316 (45.7%)
	>40 hours	375 (54.3%)
Getting a duty allowance	No	245 (35.5%)
	Yes	446 (64.5%)
Provider received work-related supervision	No	215 (31.1%)
	Yes	476 (68.9%)
Provider adherence to medical eligibility screening	Low adherence	415 (60.1%)
	Moderate adherence	118 (17.1%)
	High adherence	158 (22.9%)
Facility managing authority	Private	99 (17.7%)
	Public	461 (82.3%)
Type of facility by level	Higher-level	244 (43.6%)
	Lower-level	316 (56.4%)
Jurisdiction	Amhara	104 (18.6%)
	Oromia	140 (25.0%)
	SNNPR	88 (15.7%)
	Addis Ababa	31 (5.5%)
	Sidama	44 (7.9%)
	Emerging states	153 (27.3%)
Facility location	Urban	323 (57.7%)
	Rural	237 (42.3%)
Availability of national FP guideline	No	208 (37.1%)
	Yes	352 (62.9%)
At least one FP-trained provider is available in a facility?	No	284 (50.7%)
	Yes	276 (49.3%)
Facility has established a quality care system in place	No	172 (30.7%)
	Yes	388 (69.3%)
Facility readiness	Inadequate readiness	281 (50.2%)
	Moderate readiness	209 (37.3%)
	Adequate readiness	70 (12.5%)

### 3.2 Quality of family planning counselling

The quality of FP counselling was assessed using 15 items that captured key aspects of provider–client interaction and counselling environment. Most clients (69%) were treated respectfully, with 84% being called by their appropriate name or title. However, in nearly all consultations (97%), providers did not introduce themselves. While more than three-quarters of provider–client interactions ensured both visual and auditory privacy, only about 27% of clients were verbally assured of confidentiality. During the consultations, 42% of clients were not informed about any contraceptive methods other than the one selected. In over half of the sessions (54%), providers did not ask clients whether they had questions or concerns about the chosen method, and 62% of clients did not express any.

**Figure 1:**
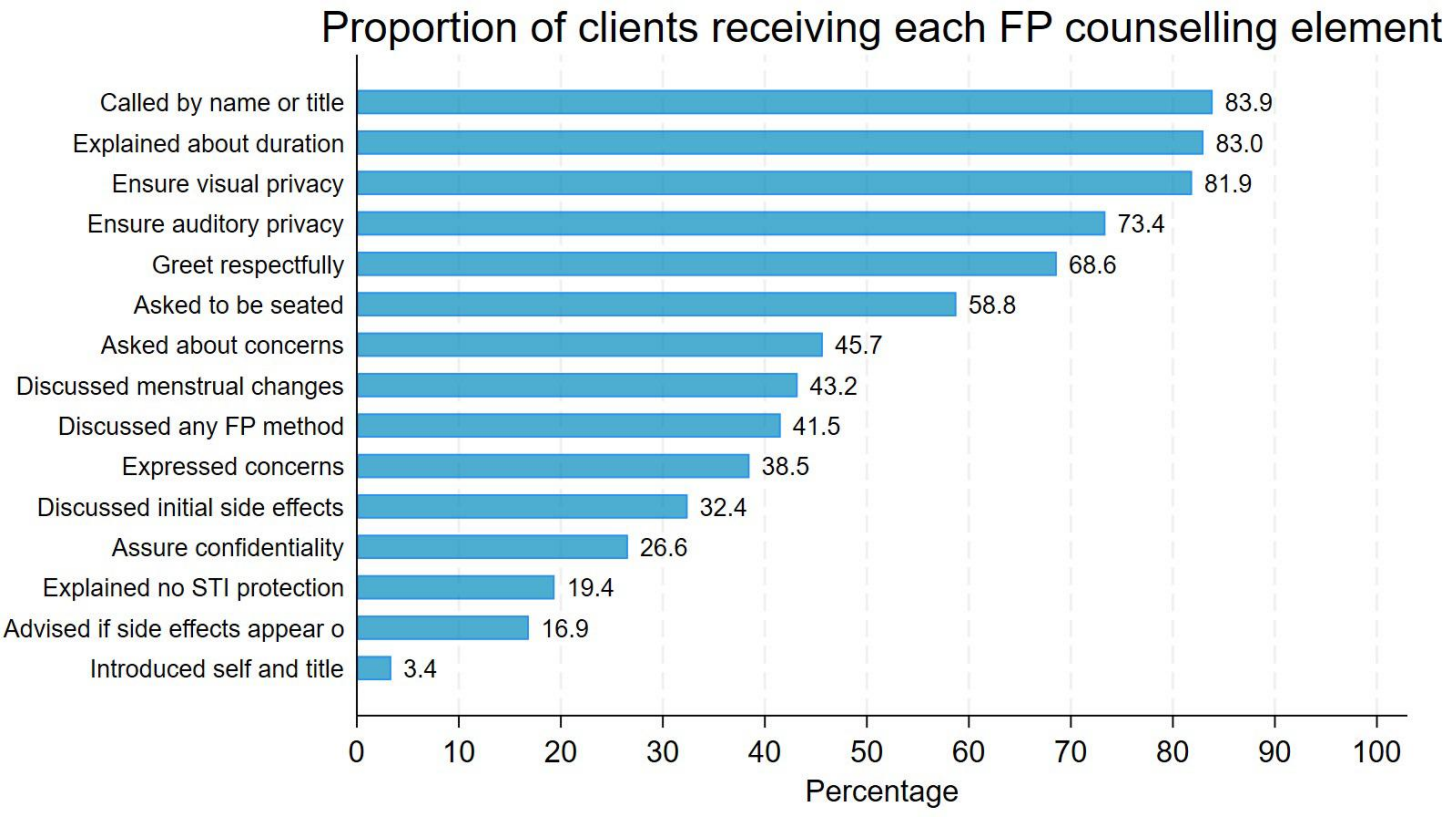
Proportion of clients who received each component of quality of family planning counselling.

Although 83% of clients were informed about how long their selected method would be effective, other essential counselling components were often missing. For example, 83% of clients were not advised on when to return if side effects occurred or persisted, 80% were not told that the method does not protect against STIs, including HIV, and 57% were not counselled about possible changes in menstruation. Overall, only 703 clients (32%) received high-quality FP counselling, while 28% received moderate-quality and 41% received low-quality FP counselling (Figure 1).

### 3.3 Predictors of quality of family planning counselling

The null multilevel ordinal logistic regression model (Model 0) revealed substantial variation in quality of FP counselling across facilities, with a facility-level variance of 4.61 and an intra-class correlation coefficient (ICC%) of 58%, indicating that over half of the total variation in counselling quality was attributable to facility-level differences.

In Model I, which incorporated client-level variables, two client-level variables were significantly associated with high-quality FP counselling. Women with secondary or higher education were more likely to receive high-quality FP counselling compared to those with no education (aOR = 1.39; 95% CI: 1.02–1.89). Similarly, compared to current FP users, both women who had used FP in the past (aOR = 1.44; 95% CI: 1.03–2.01) and those who had never used FP (aOR = 2.05; 95% CI: 1.43–2.93) were more likely to receive higher-quality FP counselling. The facility-level variance remained 4.62, and the ICC% stayed at 58%, indicating that individual client characteristics did not account for the between-facility variation.

In Model II, service provider and facility-level factors were significantly associated with the high quality of FP counselling. Providers with more than five years of experience had higher odds of delivering high-quality FP counselling (aOR = 1.81; 95% CI: 1.21–2.72). Moderate adherence (aOR = 1.66; 95% CI: 1.21–2.27) and high adherence (aOR = 4.80; 95% CI: 3.42–6.73) to medical eligibility screening were strongly associated with higher quality of FP counselling. Facilities located in Sidama (aOR = 3.20; 95% CI: 1.57–6.50) had higher odds of high-quality counselling, while those in emerging regions had significantly lower odds (aOR = 0.32; 95% CI: 0.19–0.55). The presence of at least one FP-trained provider (aOR = 1.93; 95% CI: 1.32–2.83) and the availability of national FP guidelines (aOR = 1.67; 95% CI: 1.08–2.57) were also positively associated with high-quality FP counselling. The facility-level variance in this model decreased to 3.56, with ICC% dropping to 52%, indicating that some of the facility-level differences were explained by provider and facility characteristics.

In the final Model III, which integrated all levels of predictors, all significant variables of provider and facility-level predictors from Model II, as well as women's FP status from Model I, remained significantly associated with high-quality FP counselling. However, the association with secondary and above education was no longer statistically significant in the final model. Providers with more than five years of experience were 1.91 times more likely to deliver high-quality FP counselling compared to those with two or fewer years (aOR = 1.91; 95% CI: 1.26–2.89). Providers who moderately adhered (aOR = 1.67; 95% CI: 1.22–2.29) and those who highly adhered (aOR = 4.90; 95% CI: 3.48–6.91) to medical eligibility screening questions had higher odds of high-quality FP counselling than those with low adherence.

Similarly, facilities that had at least one FP-trained provider been 1.93 times more likely to provide high-quality FP counselling than those that did not have a trained FP provider in the facility (aOR = 1.93; 95% CI: 1.31–2.85). Moreover, facilities where the national FP guideline was available were 1.70 times more likely to offer high-quality FP counselling compared to those where the guideline was not available at the time of the survey (aOR = 1.70; 95% CI: 1.09–2.64). Furthermore, facilities located in Sidama Regional State (aOR = 3.37; 95% CI: 1.63–6.98) were more likely to provide high-quality FP counselling than facilities in Oromia. Conversely, facilities located in emerging regional states were 69% less likely to provide high-quality FP counselling compared to those in Oromia (aOR = 0.31; 95% CI: 0.18–0.54).

In this final model, the facility-level variance was 3.63, with the ICC% remaining at 52%, indicating that facility-level factors continued to explain a substantial proportion of the variation in FP counselling quality. Model comparisons using log-likelihood and AIC showed that model fit improved with the inclusion of client-, provider-, and facility-level factors. Log-likelihood increased from –2098.07 in the null model to –2033.15 in the full model. Correspondingly, AIC decreased from 4202.15 to 4134.30, indicating that the full model (Model 3) achieved the best balance between model fit (Table 2).

**Table 2:** Predictors of quality of family planning counselling, Ethiopian service provision assessment survey 2021/22.

Variables	Categories	Null model	Model I aOR (95% CI)	Model II aOR (95% CI)	Model III aOR (95% CI)
Women educational status	No education		1.00		1.00
	Primary		1.03 (0.76, 1.38)		0.95 (0.71, 1.28)
	Secondary and above		**1.39** **(1.02, 1.89)**		1.23 (0.90, 1.69)
Women age in years	<25 years		0.91 (0.64, 1.31)		0.96 (0.67, 1.37)
	25–29 years		0.89 (0.65, 1.22)		0.97 (0.71, 1.33)
	30–34 years		1.00		1.00
	≥35 years		1.28 (0.82, 1.99)		1.21 (0.79, 1.89)
Women marital status	Not married		1.00		1.00
	Married		1.07 (0.69, 1.65)		0.96 (0.63, 1.48)
Parity	Nulliparous		0.95 (0.52, 1.71)		0.79 (0.44, 1.42)
	Primiparous		1.30 (0.79, 2.13)		1.22 (0.75, 2.00)
	Multiparous		1.08 (0.71, 1.64)		1.00 (0.66, 1.50)
	Grand multiparous		1.00		1.00
Women's family planning history	Current user		1.00		1.00
	Nonuser, used in past		**1.44** **(1.03, 2.01)**		**1.40** **(1.01, 1.95)**
	Nonuser, no past use		**2.05** **(1.43, 2.93)**		**2.05** **(1.44, 2.92)**
Provider age	<25 years			0.94 (0.54, 1.66	0.97 (0.55, 1.71)
	25–29 years			0.98 (0.66, 1.44	1.00 (0.68, 1.48)
	30–34 years			1.00	1.00
	≥35 years			0.68 (0.41, 1.15)	0.68 (0.40, 1.15)
Provider gender	Female			1.14 (0.84, 1.56)	1.14 (0.84, 1.56)
	Male			1.00	1.00
Provider qualification	Doctors			1.65 (0.55, 4.95)	1.78 (0.58, 5.40)
	Degree			1.00	1.00
	Diploma/HEW			0.77 (0.57, 1.06)	0.79 (0.59, 1.08)
Provider experience	≤2 years			1.00	1.00
	3–5 years			1.01 (0.70, 1.45)	1.01 (0.70, 1.45)
	>5 years			**1.81** **(1.21, 2.72)**	**1.91** **(1.26, 2.89)**
Provider working hours	≤40 hours			1.00	1.00
	>40 hours			0.77 (0.55, 1.08)	0.76 (0.54, 1.07)
Getting a duty allowance	No			1.00	1.00
	Yes			1.44 (0.97, 2.14)	1.45 (0.97, 2.18)
Provider received work-related supervision	No			1.00	1.00
	Yes			1.22 (0.86, 1.74)	1.24 (0.87, 1.77)
Adherence to medical eligibility screening	Low adherence			1.00	1.00
	Moderate adherence			**1.66** **(1.21, 2.27)**	**1.67** **(1.22, 2.29)**
	High adherence			**4.80** **(3.42, 6.73)**	**4.90** **(3.48, 6.91)**
Facility managing authority	Private			1.00	1.00
	Public			0.71 (0.37, 1.36)	0.71 (0.36, 1.37)
Type of facility by level	Higher-level			1.00	1.00
	Lower-level			0.75 (0.49, 1.17)	0.79 (0.51, 1.24)
Jurisdiction	Amhara			0.98 (0.56, 1.70)	1.06 (0.60, 1.87)
	Oromia			1.00	1.00
	SNNPR			0.97 (0.55, 1.73)	0.96 (0.53, 1.72)
	Addis Ababa			1.26 (0.54, 2.93)	1.23 (0.52, 2.93)
	Sidama			**3.20** **(1.57, 6.50)**	**3.37** **(1.63, 6.98)**
	Emerging states			**0.32** **(0.19, 0.55)**	**0.31** **(0.18, 0.54)**
Facility location	Urban			1.00	1.00
	Rural			0.89 (0.58, 1.37)	0.92 (0.59, 1.43)
Availability of national FP guideline	No			1.00	1.00
	Yes			**1.67** **(1.08, 2.57)**	**1.70** **(1.09, 2.64)**
Facility readiness in equipment and supplies	Inadequate			1.00	1.00
	Moderate			1.36 (0.89, 2.08)	1.39 (0.89, 2.13)
	Adequate			0.77 (0.42, 1.40)	0.74 (0.40, 1.37)
Having at least one provider trained in FP	No			1.00	1.00
	Yes			**1.93** **(1.32, 2.83)**	**1.93** **(1.31, 2.85)**
Quality of care system	No			1.00	1.00
	Yes			1.21 (0.72, 2.03)	1.22 (0.74, 2.05)
Random effect					
Variance		4.64	4.84	3.00	3.2
ICC (%)		59%	59%	48%	49%
Model fitness					
Log likelihood		−2,090.44	−2,075.05	−2,038.05	−1,975.08
AIC		4,186.88	4,178.11	4,031.35	4,028.17
Sample		2,224	2,224	2,232	2,224

Bold indicates statistical significance at p-value < 0.05.

## 4 Discussion

This study assessed the predictor factors of high-quality FP counselling in Ethiopia using nationally representative SPA 2021/22 data. The findings revealed that only 32% of clients received high-quality FP counselling. It also demonstrated a considerable variation in the quality of FP counselling, influenced mainly by provider and facility-level factors, with over half (52%) of the total variance attributed to facility-level characteristics.

Our findings revealed that never users and non-current FP users received significantly higher-quality FP counselling compared to women who were already using contraceptive methods at the time of consultation. Previous studies also demonstrated similar findings (20, 30). This disparity is not unexpected, as counselling approaches may differ between new and continuing users. While first-time users may require more comprehensive counselling to understand available methods and make informed decisions, current users also benefit from continued support, particularly in managing side effects or complications. Strengthening ongoing counselling for current users is essential to reducing discontinuation rates and preventing unintended pregnancies and related health risks.

The results of this study showed that providers with more experience delivered a significantly higher-quality FP counselling compared to those with less experience. This finding aligns with previous evidence suggesting that greater clinical experience enhances provider competence, communication skills, and confidence in managing client interactions (18). Over time, experienced providers are more likely to have more opportunities to receive and integrate FP-specific training into their practice, allowing them to gain a deeper understanding of counselling principles and the technical aspects of FP services. In addition, the current study indicated that providers who performed FP medical eligibility screening were more likely to provide higher-quality FP counselling than those who did not. Conducting such screenings reflects adherence to clinical guidelines and an emphasis on client safety, which are fundamental components of client-centred care. Unlike general experience alone, performing eligibility screening highlights a provider's commitment to applying technical protocols during each consultation. While this practice may be influenced by years of experience, it also suggests an active effort to follow standard procedures and maintain high-quality service delivery.

Our findings also showed that health facilities with at least one FP-trained provider were more likely to offer high-quality FP counselling compared to those without trained providers. This is likely because facilities with FP-trained staff are better equipped with the necessary skills and up-to-date knowledge essential for delivering effective and comprehensive counselling, typically gained through in-service training (27). In contrast, facilities lacking trained personnel may provide outdated or incomplete information. This underscores the critical importance of ongoing in-service training in maintaining and enhancing provider competence. It also highlights the need for both federal and regional governments to ensure that all health facilities are staffed with at least one FP-trained provider. Given the potential for staff turnover, training should be continuous, and robust monitoring and evaluation mechanisms must be established to assess the quality and impact of these training programs.

Facilities where the national FP guideline was available had significantly higher odds of providing high-quality FP counselling. A previous study using the 2014 SPA supports this finding, showing that women who received services in facilities with available guidelines reported higher satisfaction with FP services (31). This satisfaction might be attributed to receiving more comprehensive and higher-quality counselling. This finding underscores the value of supportive national guidelines, which help providers stay updated on counselling procedures and principles, and enable them to offer evidence-based, client-centred practices. At the same time, it highlights the importance of the Federal Ministry of Health (MoH) in ensuring wider distribution of national guidelines and strengthening institutional capacity and organisational readiness to deliver quality services.

Moreover, our study revealed that women who received consultation for FP at facilities in the Sidama Regional State received higher-quality FP counselling compared to those who attended FP counselling in Oromia. Conversely, women who received FP counselling from facilities located in emerging Regional States were substantially less likely to receive high-quality FP counselling compared to women who received it from facilities located in Oromia. This may be due to disparities in infrastructure, staff competencies, supervision mechanisms, and access to guidelines and commodities among the Regional States in Ethiopia (32).

Previous studies have shown that client factors, such as educational status and residence, are associated with high-quality FP counselling (16, 19, 20). However, we did not find an association between these variables and high-quality FP counselling in the current study. This difference might relate to the varying methods used to measure the quality of FP counselling or to the different variables included in the analysis. Most previous studies used the MII to measure the quality of FP counselling, which is based on three to six selected methods related questions (19, 20). In contrast, our measurement is comprehensive, comprising 15 questions.

In addition to the predictors, this study revealed a significant shortcoming in the delivery of FP counselling. While over 80% of consultations with clients ensured privacy, and clients were called by their name or title and received information about the method duration, most did not receive essential information such as potential side effects, menstrual changes, when to return if problems occurred, and assurances of confidentiality. Notably, 83% of clients were not advised to return to the facility when they experienced side effects, which may lead to discontinuation and unintended pregnancies. This lack of information can also contribute to widespread misconceptions and negative community attitudes toward contraceptive methods (8). Moreover, around 80% of clients were not informed that the methods do not protect against STIs, including HIV, representing a significant missed opportunity to promote dual protection. Given the high prevalence of STIs, including HIV, in the country (33) and ongoing conflicts, this omission may contribute to ongoing transmission risk. Supporting this concern, a study in Ethiopia indicated a higher HIV risk among contraceptive users, potentially reflecting misconceptions (33). These service gaps may result from limitations in provider training, time constraints, or inadequate focus on communication skills during counselling. Addressing these weaknesses is critical to empowering women to make informed reproductive health decisions. Strengthening service integration is also essential to ensure that FP clients receive appropriate counselling on STIs, and vice versa.

Furthermore, our study found that 73% of women had not been reassured about the confidentiality of the FP counselling and services they received. This uncertainty is especially concerning in Ethiopia, where strong socio-cultural norms, fears of monitoring, and potential repercussions discourage open discussions on reproductive health (34, 35). Women face pressure from husbands, in-laws, and communities to have large families, and concerns about confidentiality can deter them from seeking services or sharing sensitive information (35, 36). Without assurances of confidentiality, some may resort to secret contraceptive use, increasing the risk of inconsistent use and health complications due to a lack of follow-up (37, 38). Therefore, reinforcing confidentiality in FP counselling is not only an ethical obligation but also a crucial strategy to build trust, address cultural barriers, and improve service uptake and continuation, ultimately empowering women in their reproductive choices.

### 4.1 Strengths and limitations

A key strength of this study is the utilisation of a nationally representative dataset comprising both public and private facilities as well as all levels of facilities, which facilitates the robust generalisability of the findings across the Ethiopian health system. Additionally, the data for this study were collected through facility inventory, health worker interviews, direct observation, and exit interviews, which enabled us to obtain comprehensive information from client, provider, and facility-level factors that could influence the quality of FP counselling and minimise recall bias. Furthermore, the multilevel modelling approach allowed for the partitioning of variance at the client, provider and facility levels, providing nuanced insights into the predictors of counselling quality.

However, several limitations should be noted. First, the cross-sectional nature of the data prevents causal inference. Second, the SPA data did not include the Tigray regional state due to security concerns, which may limit the full national generalisation. Third, while trained observers and exit interviews can help reduce recall bias and collect more accurate data, the potential for the Hawthorne effect cannot be entirely ruled out. Fourth, although the study accounted for a wide range of predictors, unmeasured confounders, such as provider motivation, client wealth status or occupation, etc., were not considered in our analysis due to their absence in the dataset. Finally, while the checklist items addressed key aspects of quality, certain nuances of client-centred communication and non-verbal provider behaviour may not have been adequately captured.

## 5 Conclusion

This study found that only a minority of FP clients in Ethiopia received high-quality counselling, with substantial variation across facilities, explained mainly by provider and facility-level factors. Client FP status, provider experience, adherence to medical eligibility screening, the presence of trained providers, availability of national FP guidelines, and regional context were significant predictors of FP counselling quality. Additionally, this study revealed that women received limited information, particularly regarding the selected method, including when to return to the facility if side effects persist, what to do when menstrual changes occur, and the method does not prevent STIs, including HIV.

Improving the quality of FP counselling would benefit from regular in-service training and mentorship—especially for newly deployed or less-experienced providers—which would help strengthen their skills and build confidence. Promoting better communication practices, such as clear provider introductions, providing assurance about confidentiality, and offering comprehensive method-specific guidance, would also enhance client trust and understanding. Ensuring the availability and routine use of national FP guidelines across all facilities, particularly in underserved and remote areas, would help standardise service delivery and promote evidence-based practices. Additionally, targeted investments in emerging regions would be beneficial in addressing the disparities in the quality of FP counselling. Moreover, while providing comprehensive counselling to new clients is essential and should be strengthened, ongoing counselling for current users would help address their concerns, reinforce understanding of side effects, and significantly reduce discontinuation. Future qualitative research would be valuable for exploring the perspectives of both clients and providers, thereby deepening the understanding of barriers to high-quality counselling and informing the design of responsive, context-appropriate interventions.

## Data Availability

Publicly available datasets were analyzed in this study. This data can be found here: https://www.DHSprogram.com.

## Appendix Table 1 Definition and categorisation of independent variables.

**Table:**

Category	Variable	Definition/Categorisation
Client-related	Age	<25 years, 25–29 years, 30–34 years, ≥35 years
	Marital status	Currently married/living together vs. not married (divorced/separated, widowed, never married)
	Educational attainment	No education, primary, secondary and higher education
	Parity	Nulliparous (0), primiparous (1), multiparous (2–5), grand multiparous (>5)
	FP status at consultation	Current user, past user, or never user
Provider-related	Age	<30 years, 30–34 years, ≥35 years
	Gender	Female or male
	Professional qualification	Doctors (GPs, specialists, IESO), degree-level (health officers, nurses, midwives), diploma-level (nurses, midwives, HEWs)
	Work experience	≤2 years, 3–5 years, >5 years
	Weekly working hours	≤40 h vs. >40 h
	Salary supplement (duty allowance)	Yes or no
	Supervision received	Yes (any supervision in last 12+ months) vs. no (no supervision)
	Adherence to medical eligibility screening	Assessed based on six recommended clinical practices: measuring blood pressure, weighing the client, asking about smoking habits, inquiring about chronic conditions, asking about symptoms of sexually transmitted infections, and asking about current medication use. Each action was coded as 1 if it was performed and 0 if it was not. The total adherence score ranged from 0 to 6 and was categorised into low adherence (0–2 actions), moderate adherence (3–4 actions), and high adherence (5–6 actions).
Facility-related	Facility type	Higher-level (tertiary/general/primary hospitals) vs. lower-level (health centres/posts/clinics)
	Managing authority	Public or private
	Facility location	Urban or rural
	Jurisdiction	Categorised based on the regional state structure at the time of the survey. Regions were classified into: Amhara, Oromia, Southern Nations, Nationalities, and Peoples' Region (SNNPR), Sidama, and Addis Ababa City Administration. All other regions—Afar, Benishangul-Gumuz, Dire Dawa City Administration, Gambela, Harari, and Somali—were grouped under emerging regional states. Tigray Regional State was not included in the survey due to the ongoing civil war during the data collection period.
	Availability of national FP guideline	Yes or no
	Number of FP-trained providers in the last 24 months	Binary: yes (≥1 trained) or no (none trained)
	Quality assurance system	Facilities were asked whether they had a system in place to ensure service quality. Original responses included observed systems, reported systems, systems without maintained records, no system, and “don't know”. For analysis, this variable was recoded into “yes” (if any form of QA system was reported or observed) and “no” (if no QA system was reported). Facilities with “don't know” responses were excluded from the analysis.
	Facility readiness index	Constructed using a composite score based on the observed availability of seven essential items for FP counselling: a blood pressure apparatus (manual or digital), a source of examination light, a male condom demonstration model, samples of FP methods, a pelvic model for IUD demonstration, visual aids (e.g., flip charts or leaflets), and an examination couch or bed. Each item was scored 1 if available and 0 if not. The total score ranged from 0 to 7 and was categorised as inadequate readiness (0–2 items), moderate readiness (3–5 items), and adequate readiness (6–7 items).
